# Analytical Investigation of the Possible Chemical Interaction of Methyldopa with Some Reducing Carbohydrates Used as Pharmaceutical Excipients

**DOI:** 10.15171/apb.2018.074

**Published:** 2018-11-29

**Authors:** Mohammad Reza Siahi, Soma Rahimi, Farnaz Monajjemzadeh

**Affiliations:** ^1^Drug Applied Research Center, Tabriz University of Medical Sciences, Tabriz, Iran.; ^2^Department of Pharmaceutical and Food Control, Tabriz University of Medical Sciences, Faculty of Pharmacy, Tabriz, Iran.; ^3^Student Research Committee, Tabriz University of Medical Sciences, Faculty of Pharmacy, Tabriz, Iran.; ^4^Food and Drug Safety Research Center, Tabriz University of Medical Sciences, Tabriz, Iran.

**Keywords:** Pharmaceuticals, Thermal Methods, IR Spectroscopy, LC-Mass analysis, Incompatibility, Excipients

## Abstract

***Purpose:*** Assessment of drug substance and excipients compatibility is an important issue during pre-formulation studies as well as the quality control of pharmaceutical dosage forms. In this study, potential incompatibility between methyldopa and reducing excipients was evaluated using physicochemical methods.

***Methods:*** Dextrose and lactose (anhydrous & monohydrate) were selected as reducing carbohydrates. The initial incompatibility was studied with DSC and FTIR on binary mixtures with 1:1 mass ratio. Results were confirmed using HPLC studies coupled with mass spectrometry.

***Results:*** The DSC curves indicated the elimination of the melting endotherm of methyldopa in the binary mixtures. A new peak at 1719 cm-1 was observed in the FTIR spectra that can be attributed to the loss of type one amine functionality. The m/z of the proposed compound was observed in the mass spectra.

***Conclusion:*** The potential incompatibility of Methyldopa with reducing carbohydrates was established using physicochemical methods.

## Introduction

The study of incompatibilities between the drugs and excipients is an important part of pre-formulation stage and provides important information to reach a suitable formulation.^1^

Most excipients have no direct pharmacological action, but they can involve physical and chemical interactions with active pharmaceutical ingredients.^1,2^

Methyldopa (3-Hydroxy-α-methyl-l-tyrosine sesquihydrate) is an alfa2 agonist used as a central anti-hypertensive as well as an anti-Parkinson agent. Methyldopa is the first choice treatment in pregnancy induced hypertension (PIH).^3^ This medication has been listed on the World Health Organization's List of Essential Medicines.^4^

The Maillard reaction is one of the most common incompatibility interactions between the amine containing drug molecules and reducing excipients.^5^ Briefly, the reactive carbonyl group in the reducing carbohydrate reacts with the nucleophilic amine moiety of the drug moiety.

Methyldopa has a type 1 amine functional group in this structure, and thus is a candidate for the nucleoiphilic interaction with reducing agents. Dextrose and lactose (anhydride & monohydrate) used in pharmaceutical dosage forms for various purposes were selected as reducing carbohydrates. In solid pharmaceutical dosage forms, lactose is used as a filler in conventional tablets and capsules, as a carrier in liquisolid formulations, and as a lyoprotectant in lyophilized sterile or non-sterile powders.^6,7^ Dextrose is also used as a binder in the granulation of the conventional dosage forms. It is also used as a sweetener in liquid and sugar coated solid dosage forms. It is worthy to mention that 5% dextrose large volume intravenous fluids (LVIFs) may be used to prepare gavage solutions of special drugs to be administered via gavage route (enteral tubing) mainly in unconscious patients and in experimental studies on laboratory animals by crushing the marketed tablet dosage forms and dissolving in LVIFs.^7-10^ To the best of our knowledge, no previous evaluation has been conducted in this case; therefore, the potential incompatibility between methyldopa and reducing excipients will be evaluated using physicochemical methods.

## Materials and Methods

Methyldopa ((S)-2-amino-3-(3,4-dihydroxyphenyl)-2-methyl-propanoic acid) was obtained from Dipharma Francis Pharmaceutical Co. (Baranzate, Italy). Monohydrate and anhydrous lactose were provided from DMV Chemical Co. (Veghal, Netherlands). Acetonitrile and formic acid were purchased from Merck (Darmstadt, Germany). Magnesium sulfate, heptane sulfonic acid, sulfuric acid, methylene chloride, dimethyl formamide, acetic acid and all other chemicals were obtained from Labscan Analytical Science (Dublin, Ireland). Generic preparation of Methyldopa was acquired from local pharmacies in Iran. TLC (Thin Layer Chromatography) plates (Kieselgel GF 254 plates, 20 x 20 cm, 1 mm thick) were purchased from Merck, Germany.

### Thin Layer Liquid Chromatography (TLC)

Practical details of the TLC method was as described elsewhere.^11,12^ A mixture of ethyl acetate and methanol (1:3 v/v) containing 0.25% v/v glacial acetic acid was used as the mobile phase. Lactose with a concentration of (1mg mL^-1^) in diluent solution (methanol: water (2:3 v/v)) was spotted as the reference standard. Twenty units of brand tablets were weighed, and the mean weight was calculated. Assuming that the entire excipient content of the average weight was lactose, an equivalent of 25 mg lactose in powdered tablets content was transferred to a 25-mL volumetric flask and was diluted to 1 mg mL^-1^. Standard and test solutions (2µL) were spotted on a thin-layer chromatographic plate individually.

The spots were dried and placed in a separation chamber, which was previously saturated with the solvent. The plate was removed from the chamber before the solvent front reached the top of the stationary phase. It was dried with a stream of hot air, sprayed uniformly with staining solution containing 0.5g thymol in 95mL alcohol and 5mL sulfuric acid. Later, the plate was heated at 130 °C for 5 min., the presence of lactose was approved when the main spot resulted from these brands were similar to the standard solution in appearance and R_f_ (Retention Factor) values.

### Differential Scanning Calorimeter (DSC)

DSC curves were obtained in a differential scanning calorimeter (DSC-60, Shimadzu, Japan) using sealed aluminum pans with approximately 5mg of samples, including pure drug, pure excipient and their physical mixture in 1:1 mass ratio.^13^ Samples were homogenated thoroughly using the tumbling method. The samples were scanned at 15° C/min heating rate at the temperature range of 30- 450. TA-60 software (Version 1.51) was used for enthalpy and peak temperature calculations.

### Fourier Transform-Infrared Spectroscopy (FT-IR)

Methyldopa and excipients were blended in 1:1 mass ratios. They were mixed with 20% (v/w) water and were stored in closed vials at 70 °C for 24 hours. The controls were made using pure components and the mixture with no added water.

FTIR spectra were obtained from all samples immediately after mixing and after incubation at elevated temperatures at predetermined time intervals using the potassium bromide disc preparation method (Bomem, MB-100 series, Quebec, Canada).

### N-Formyl Methyldopa Synthesis

N-Formyl methyldopa was synthetized according to the method proposed by Mateusz et al..^14^ Briefly, drug molecule was dissolved in dimethylformamide and refluxed at 150 °C for 2 days. The resultant was filtered, and liquid –liquid extraction was performed using methylene chloride-water. After dehydration process by anhydrous magnesium sulphate, the organic layer was evaporated, and the resultant solid powder was named N-formyl methyldopa. This substance was identified using FTIR and Mass spectroscopy.

### High Pressure Liquid Chromatography (HPLC)

The HPLC analysis was performed on a cecil 100 series HPLC (Cambridge, UK) consisted of an on line degasser, CE-1100 HPLC pump and a Cecil CE-1100 variable-wavelength UV detector. Chromatograms were recorded and further analyzed using a “Data Control” Version 5.10. Computer software.

A C18 column (250 mm, 4.60 mm, 5 µm; Waters, USA) maintained at ambient temperature was used as the stationary phase. The main idea of separation was derived from Rembischevski and Gemal.^15^ Mobile phase consisted of Methanol: Buffer at 19:81 v/v ratio. Buffer was prepared as a 20% v/v acetic acid in deionized HPLC water containing 5mM of heptane sulfonic acid as an organic modifier. The detection was performed at 280 nm with 1.5ml.min^-1^ flow rate.

### Mass Spectroscopy

Mass analysis was performed on the Waters 2695 (Milford, Massachusetts, USA) Quadrupole Mass system, at electron-spray ionization mode, positive ionization, capillary voltage 3.5 V, cone voltage 60 V, extractor voltage 3 V, RF lens voltage 1V, source temperature (80°C) desolation temperature (150 °C), desolation gas flow( 350 L h^-1^) and cone gas flow (50 L h^-1^).

Mass spectra were obtained from mixture of methyldopa with dextrose and lactose blended in 1:1 mass ratio then mixture with 20 %(v/w) water and stored in the 90 °C for 5 days. Each sample powder was dissolved in a mixture of methanol: water solvent (50:50) to the approximate concentration of 100 µg mL^-1^and injected to mass detector. Mass resolution was calculated by dividing the peak intensity into peak width at the half of the maximum peak height.

### Statistical Analysis

Means and standard deviations and all other calculations were performed using Excel 2013 software.

## Results and Discussion

Although different drug to excipient ratios can be utilized in compatibility studies, in the case of fillers such as lactose. it is common to use 1:1 drug to excipient ratio.^16^ This ratio is very common in studying possible interaction products regardless of the excipient type.^14,16^

### Thin Layer Chromatography (TLC)

**Figure 1:**
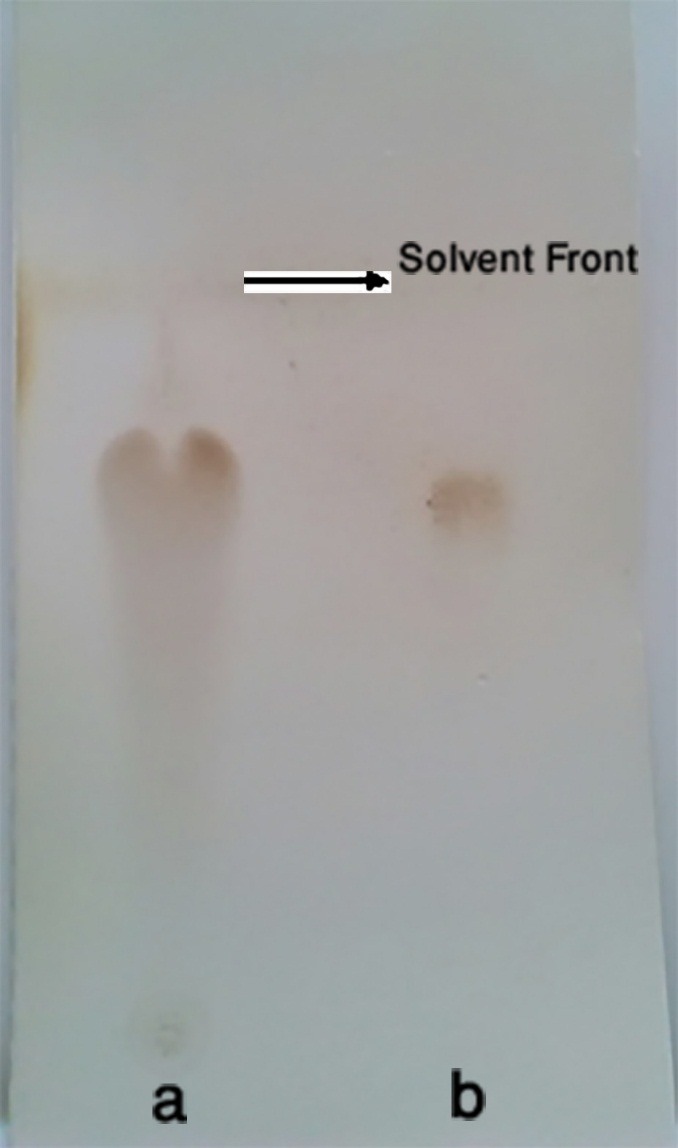
Digital imaging of the TLC plate, a) Standard and b) Brand tablet formulation

Figure 1 shows TLC results as a digital image. The (b) and (a) spots represent standard lactose and the brand samples (Figure 1).

The calculated R_F_ values for standard lactose and internal brand were 0.74 ± 0.03 and 0.77 ± 0.01, respectively. The resulted R_f_ values were in a good agreement with each other (T- Test, P-value>0.05), indicating the presence of lactose as an excipient in a sample brand. Although maillard incompatibility in pharmaceutical dosage forms has gained considerable attention in recent years, pharmaceutical industries still utilize this reducing excipient in the manufactured formulations.^16-25^

### DSC results

**Figure 2:**
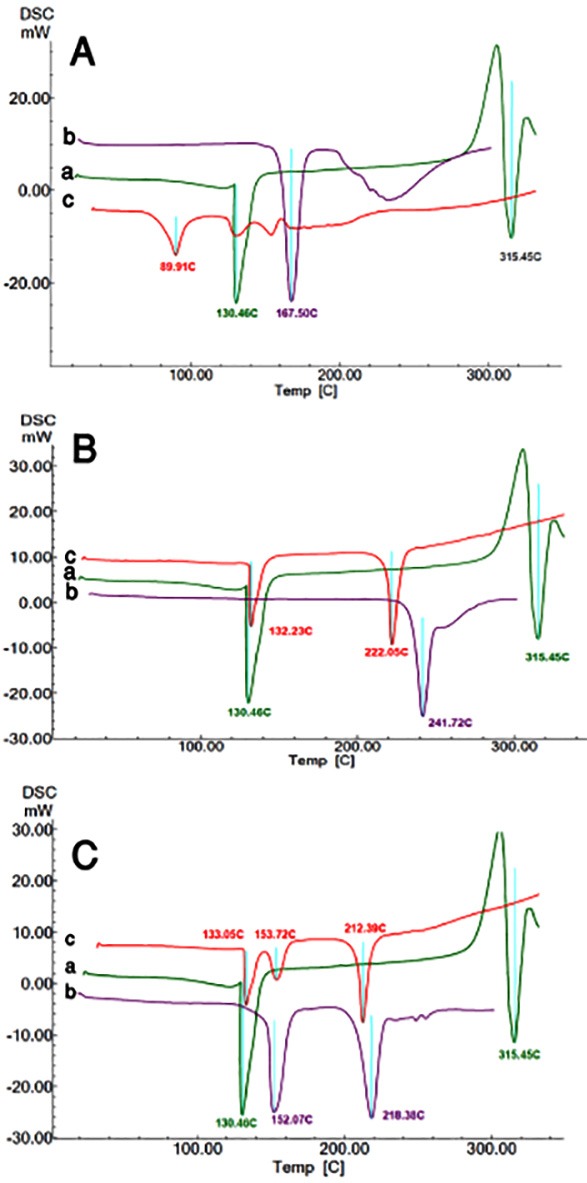
The DSC curves of drug with different excipients, A) Dextrose, B) Anhydrous Lactose and C) Monohydrated lactose, a) pure methyldopa, b) pure excipient and c) binary mixture of methyldopa and each excipient in a 1:1 w/w ratio

Figure 2A shows the DSC curves of pure dextrose, pure methyldopa and the mixture of methyldopa and dextrose.

Pure methyldopa (Figure 2A-a) showed an endothermic peak at 130.46 °C corresponding to the loss of water from the crystalline structure. The exothermic event followed by an intense endothermic peak was observed at approximately 315.45°C, being assigned to the melting point of methyldopa.^26^ The exothermic event along with melting may be due to simultaneous vaporization of the drug molecule upon melting.

Pure dextrose (Figure 2A-b) indicated an endothermic melting peak at 167.50 °C. The mixture of methyldopa and dextrose (Figure 2A-c) showed a new peak at 89.91°C, and the methyldopa melting event was disappeared completely. Since the peak of crystalline water loss can be observed at approximately 130 °C, the new peak at 89.91°C may be attributed to the water elimination of methyldopa and dextrose reaction. The lack of drug molecule melting event is another sign of drug-excipient incompatibility.

Figure 2B presents the DSC curve of binary mixture of methyldopa and anhydride lactose.

Pure anhydride lactose (Figure 2B-b) showed an endothermic melting peak at 241.72 °C. In the drug-excipient mixture (Figure 2B-c), the endothermic peak of methyldopa was eliminated and the crystalline structure water loss slightly shifted and appeared at 132.23 °C. Again, lack of drug molecule melting endotherm proposes an incompatibility.

Figure 2C shows the DSC curves of binary mixture of methyldopa.

Pure monohydrate lactose (Figure 2C-b) showed an endothermic melting peak at 218.38 °C and an endothermic water loss at 152.07°C corresponding to the water of monohydrate lactose. In the binary mixture of methyldopa and monohydrate lactose, the endothermic peak of drug melting is again missing, indicating a kind of possible incompatibility.

As previously shown, the crystalline structure of molecules (drug and or excipient) loses the water of crystallization at temperatures higher than 100 °C , while free water which can be formed due to Maillard reaction can be seen at much lower temperatures. In the three drug-excipient mixtures, water formation signs were only seen in dextrose and drug mixtures.

It should be noted that small temperature shifts toward lower values may be observed due to the amount of the materials inside a certain sample size.

### FT-IR Results

**Figure 3:**
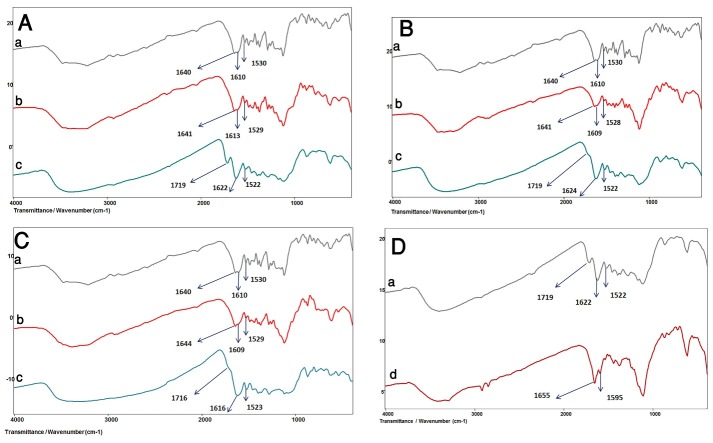
The IR spectrums, A) Drug-Dextrose, B) Drug-Anhydrous lactose, C) Drug-Monohydrated lactose, binary mixtures **a)** Pure Drug, **b)** Mixture immediately after mixing and **c)** After incubation at 90 °C after 2 months and D) Comparison of **a)**incubated Drug-Dextrose mixture with **b)** synthetized N-formyl of methyldopa

Figure 3-A-a shows the IR spectrum of pure methyldopa.

According to the literature, the main IR signals appeared at 1640cm^-1^ (carbonyl stretch in carboxylic acid with an adjacent amine), 1530cm^-1^ and 1610 cm^-1^ (Primary amine N-H bending ), 1489 cm^-1^ (Aromatic C=C), 1122 cm^-1^ (C-N stretch), 1286 cm^-1^ (R-Alkyl O-H deformation and C-O stretching vibration interaction), 1255 cm^-1^ R-Alkyl) and1119 cm^-1^ ‏)C-O of phenolic OH).

Figure 3-A(a-c) presents the IR spectrum of pure methyldopa, mixture of methyldopa and dextrose ‏)1:1 mass ratio) with 20% (v/w) added water, immediately after mixing and after incubation at 90 °C after 2 months.

Some changes in the IR absorption pattern were observed in the incubated physical mixture. The amine functional group in the chemical structure of the intact drug molecule was identified by N-H absorption peaks at (strong 1530 ,weak 1610 cm^-1^), while the carboxylic acid functionality absorbed the IR spectrum at approximately 1640 cm^-1^. After incubation, the sample showed 1522 cm^-1^absorption peak, but the absorption at 1610 cm^-1^was eliminated 3-A-c, and new and strong absorptions in 1622 and 1719 cm^-1^occurred, which may be to loss of type one amine as the result of conversion to type 2 and carboxylic acid functionality, respectively.^27-29^ The shift observed for the absorption wave number of the carboxylic acid may be due to adjacent amine change and subsequent reduced bond electro negativity.

Figures 3-B and 3-C show the IR spectrum of pure methyldopa, mixture of methyldopa with anhydrous lactose or Monohydrated lactose (1:1 mass ratio) with 20% (v/w) added water, immediately after mixing and after incubation at 90 °C after 2 months.

Similar to dextrose-drug mixtures, in the incubated physical mixtures of anhydrous lactose and monohydrated lactose (3-B-c and 3-C-c), the absorption peaks at 1522 cm^-1^remained almost unchanged, but the peak at 1610 cm^-1^was eliminated, and new and strong absorptions at approximately 1624 and 1719 cm^-1^occurred. The same conclusions made for dextrose can explain these changes.

In the incubated binary mixture of the drug with monohydrated lactose, some conversions occurred in the peak of N-H (1530, 1610 cm^-1^) and carboxylic acid (1640 cm^-1^). The maintenance of 1522 cm^-1^and elimination of 1610 cm^-1^were seen, and a new absorption in 1622 and 1719 cm^-1^occurred, which may be to loss of type one amine due to conversion to type 2 (Figure 3-C-c).

The findings indicated that the newly formed peak at 1719 cm^-1^ was more intense in dextrose binary mixtures with methyldopa compared to lactose samples.

Figure 3-D illustrates the chemically synthetized N-formyl methyldopa IR absorption spectrum along with the most reacted sample mixture (dextrose-Methyldopa binary mixture).

The N-formylated methyldopa IR spectrum (Figure 3-D-b) depicts the elimination of the IR absorption bond in 1700 cm^-1^, and formation of a new peak in 1655 cm^-1^ corresponded to bending of C-O in N-formyl or type 2 amide functionality. The shape of the reaction mixtures (Figure 3-D-a) compared to N-formyl methyldopa indicates that the reaction progress is intermediate and no N-formylated methyldopa as an end stage product is formed yet.

### HPLC results

**Figure 4:**
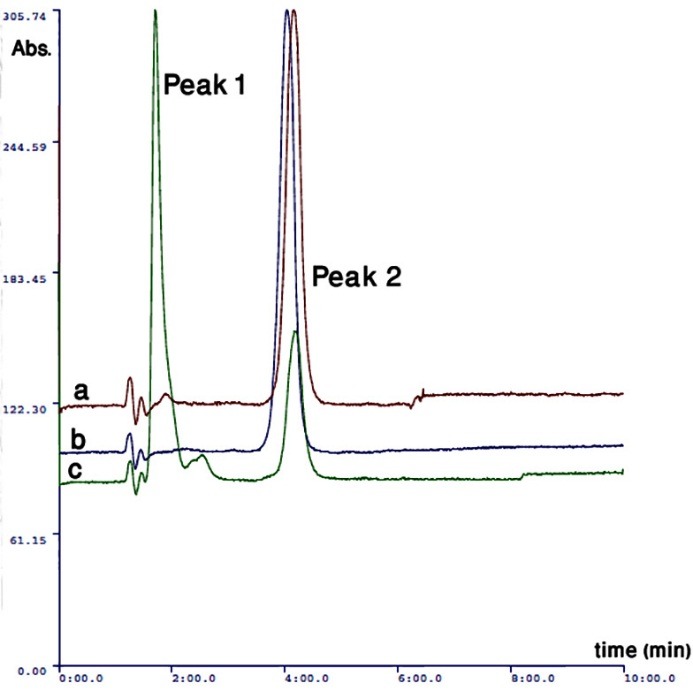
The HPLC chromatogram of, a) Drug-Anhydrous lactose binary mixture, b) Pure drug and C) Drug-Dextrose binary mixture

Figure 4 shows HPLC chromatogram of standard methyldopa, binary mixtures of methyldopa with anhydrous lactose or dextrose (1:1 mass ratio).

According to Figure 4, Peak 1 is a new peak observed in binary mixtures and is more intense in the case of Drug-Dextrose binary mixture. The peak 1 was collected from the exit part of the column after uv detector and was consequently injected to the mass analyzer.

### Mass results

**Figure 5:**
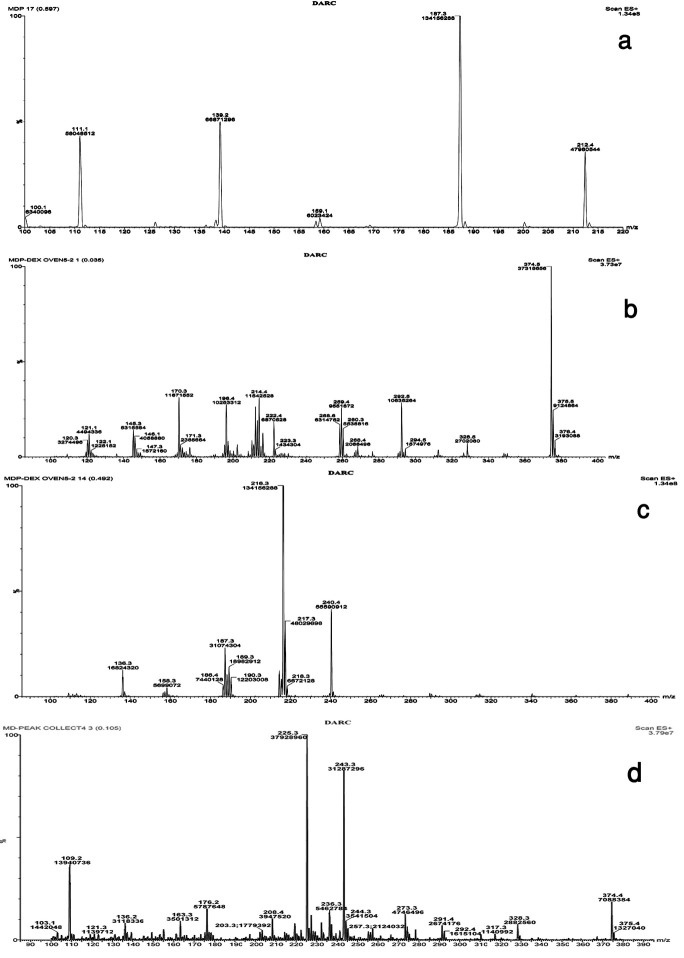
The mass spectrum of a) Methyldopa, b) Methyldopa-Dextrose binary mixture at 90 °C for 5 day, c) Methyldopa-Dextrose binary mixture at 60 °C for 12 months

**Figure 6:**
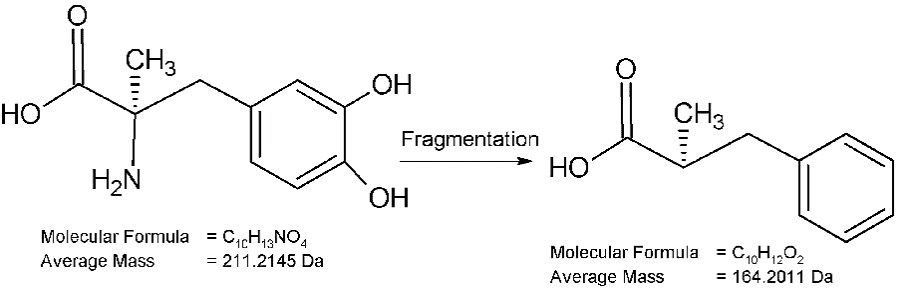
Fragmentation pattern of methyldopa

Figure 5-a presents the mass spectrum of methyldopa as a reference standard powder. The m/z value at 212.4 corresponds to the‏ [M+H^+^] of the molecular ion of the methyldopa (Molecular weight=211.2). Figure 6 shows the proposed fragmentation pathway of methyldopa resulting in an m/z value of 164, which increases to 187.2 by accepting a Sodium ion and leads to the base peak showing the highest intensity at m/z value equal to 187.3 (Figure 4-a).

**Figure 7:**
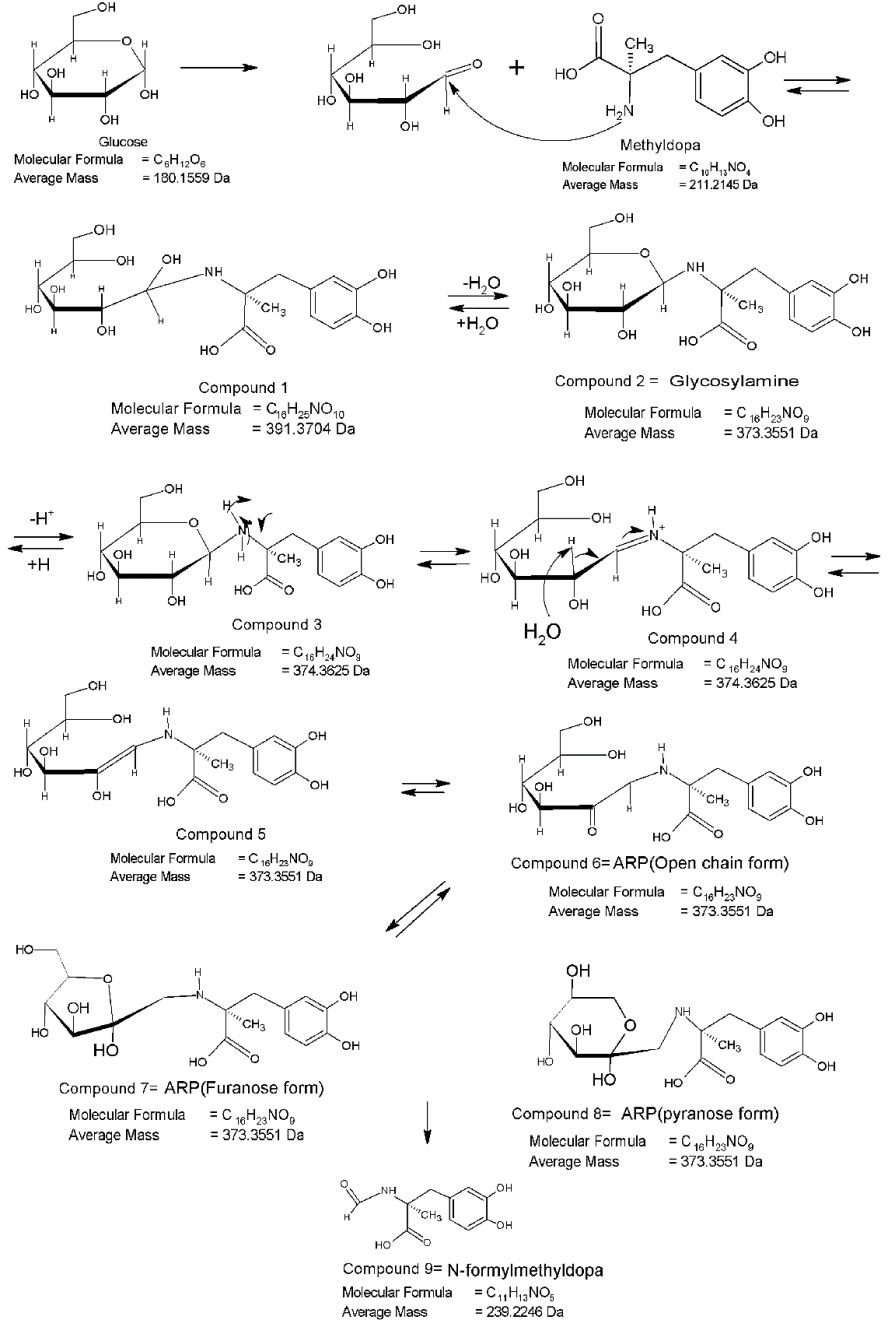
The proposed interaction pathway of methyldopa with dextrose

Figure 7 shows the proposed interaction pathway of methyldopa with dextrose.

Figures 7-b and 7-c present the mass spectrums of the methyldopa and dextrose mixture stored at 90 °C for 5 day or 60 °C for 12 months, respectively.

According to Figure 7, it is interesting to note that all the compounds numbered 2,5,6,7 and 8 are structural isomers formed by rearrangement reactions. These isomers are referred as intermediate products herein. Thus, the [M+H^+^] peaks of these isomeric structures appear similarly in m/z values of approximately 374.3, while the N-formyl compound referred to as end stage Maillard reaction product herein, may appear at m/z value equal to 240.2.

Dextrose containing samples showed the intermediate and end stage (N-formyl) reaction products at m/z values equal to 374.5 and 240.4, respectively (Figures 5-b and 5-c). It can be concluded that in drug-Dextrose binary mixtures, the end stage products are formed under more intense condition compared to the intermediate ones.

**Figure 8:**
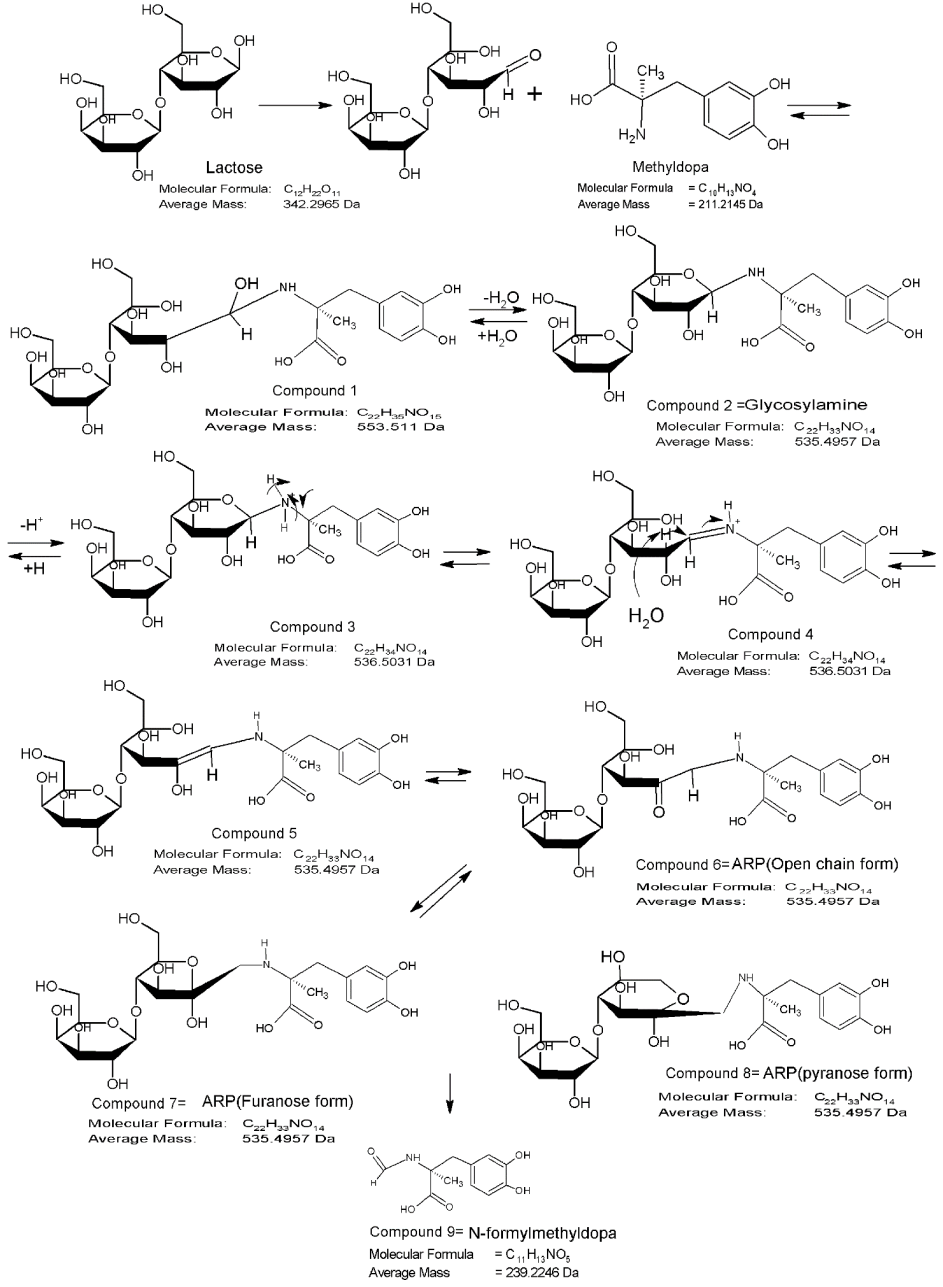
The proposed interaction pathway of methyldopa with lactose anhydride

Figure 8 presents the proposed interaction pathway of methyldopa with lactose.

**Figure 9:**
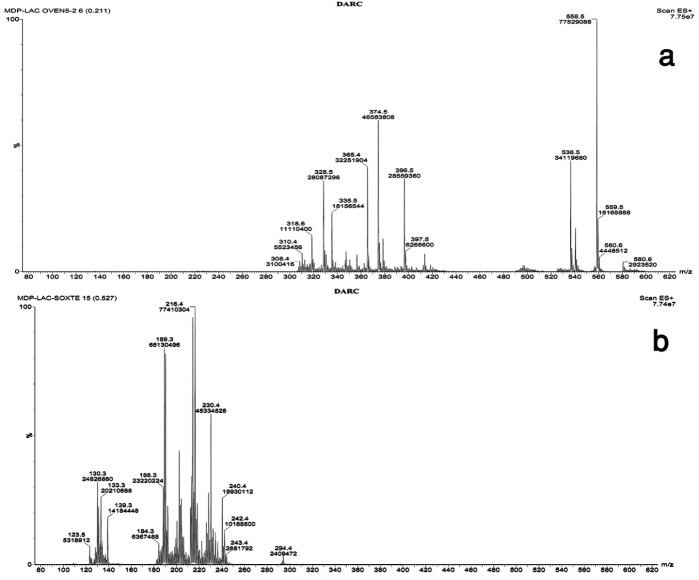
The mass spectrum of a) Methyldopa-Lactose binary mixture stored at 90 °C for 5 days, b) Methyldopa- Lactose binary mixture at 60 °C for 12 months

Figure 9 depicts the mass spectrum of Methyldopa-Lactose binary mixture stored at 90 °C for 5 days and at 60 °C for 12 months.

Figure 9-a shows the mass spectrum of methyldopa-anhydrous lactose mixture stored at 90 °C for 5 days. The base peak appeared at m/z value equal to 558.5, corresponding to the [M+H^+^] of compound 2 containing one sodium ion. Meanwhile, the m/z value of compound 2 with no sodium is also obvious in the mass spectrum at 536.5 (Figure 9-a). A close look at chemical structures of compounds 2,5,6,7 and 8 indicates that all these compounds are structural isomers and thus their [M+H^+^] ion peak appears similarly at approximately 536.5. In the 5-day stored Drug-Lactose mixture, there was no sign of formation of end stage (N-formyl) product (Figure 9-a), while the long time treated sample for one year showed a strong peak at m/z value equal to 240.4 (Figure 9-b).

Figure 8 shows the mass spectrum of mixture of methyldopa and anhydride lactose blended in 1:1 mass ratio and then mixed with 20% (v/w) water and stored at 60 °C for 1 year. In this spectrum, the m/z of N-formyl (240.4) is clearly observed. It can be concluded that in drug-lactose binary mixtures, the end stage products are formed under more intense condition compared to the intermediate ones.

## Conclusion

The potential incompatibility of Methyldopa with reducing carbohydrates was successfully established using simple and sophisticated physicochemical methods.

It is recommended to track the Maillard interaction in solid pharmaceutical dosage forms containing lactose as a filler (for example in tablets and capsules), or as a carrier in liquisolid formulations, and or as a lyoprotectant in lyophilized sterile or non-sterile powders ^6,7^. The same is correct for Dextrose containing preparations used as a binder in the granulation and as a sweetener in liquid and sugar coated solid dosage forms. In the case of Dextrose-Methyldopa interaction, the 5% dextrose large volume intravenous fluids (LVIFs) that may be used as gavage solution diluents in enteral tubing of unconscious Parkinson patients may trigger the unwanted interaction, and may be simply avoided or tracked using the introduced physicochemical methods.^7-9^

## Ethical Issues

Not applicable.

## Conflict of Interest

The authors have no conflict of interests.

## Acknowledgments

This paper was extracted from Pharm.D thesis no. 3801 submitted to the Faculty of Pharmacy, Tabriz University of Medical Sciences and financially supported by a grant from the Drug Applied Research Center of the same university. We express our thanks to Dr. Hadi Hamishehkar for providing the gavage information clinically.
